# In this month’s Bulletin

**DOI:** 10.2471/BLT.17.000817

**Published:** 2017-08-01

In the editorial section, Aziz Sheikh et al. (546) announce WHO's third Global patient safety challenge on reducing medication-related harm. Adilya Albetkova et al. (547) argue for specialized training of laboratory directors so that national governments can meet their obligations under the *International Health Regulations (2005)*.

Sophie Cousins (550–551) reports on the difficulties faced by national diabetes prevention and treatment programmes in WHO's South-East Asia region. Andréia Azevedo Soares (552–553) interviews Sally J Rogers about evidence-based approaches to young children with autism.

## China

### Fast disappearing

Shengjie Lai et al. (564–573) analyse malaria trends and programme costs from 2011-2015.

## China, Philippines, South Africa, Swaziland, United States of America 

### Where to treat multidrug-resistant tuberculosis?

Jennifer Ho et al. (584–593) collate the evidence for effectiveness of decentralized care.

## Ghana

### Small babies, difficult course

Maureen O'Leary et al. (574–583) study low birth weight as a risk factor.

## South Africa

### Impact of a rapid test

Sabine Hermans et al. (554–563) report a decrease in empirical treatment for tuberculosis. 

## Thailand

### Joining forces on antimicrobial resistance

Viroj Tangcharoensathien et al. (599–603) chart a path from global agenda to national health and agricultural actions.

## Uganda

### Dealing with expired stocks

Pakoyo Fadhiru Kamba et al. (594–598) assess the downsides of pharmaceutical donations.

## Global 

### Improving health of women, adolescents and children

Carlos Dora et al. (604–607) argue that environmental health policies can impact health outcomes. 

### Vector control options

Steve W Lindsay et al. (606–608) consider the spread of arboviruses and vector control measures.

